# Aberrant bone metabolism in critical illness

**DOI:** 10.1186/cc10757

**Published:** 2012-03-20

**Authors:** I Vanhees, L Solie, SJ Roberts, A Wauters, J Gunst, F Luyten, S Van Cromphaut, G Van den Berghe, HC Owen

**Affiliations:** 1 Katholieke Universiteit Leuven, Belgium

## Introduction

Critically ill patients present with distinct alterations in bone metabolism. We have previously reported a decrease in bone formation markers and a dramatic increase in bone resorption markers. In a rabbit model of critical illness, we observed significantly lower bone mineral content in the trabeculae of critically ill rabbits compared to healthy controls. This suggested uncoupling between bone formation and degradation during critical illness, and could increase risk of fracture during rehabilitation or impaired healing of bone fractures. In this study, we investigated the effect of critical illness on bone metabolism at the tissue and cellular level.

## Methods

Circulating CD14/CD11b osteoclast precursors in peripheral blood samples of critically ill patients and healthy controls were measured by flow cytometry. Peripheral blood mononuclear cells (PBMCs) were isolated and differentiated towards osteoclasts *in vitro *in 10% healthy (HS) or patient serum (PS) for 14 days. When analyzing bone formation, human periosteal-derived cells (hPDCs) were cultured *in vitro *in 10% HS or PS, and analyzed for osteoblast differentiation after 14 days. Bone formation was studied using serum-treated hPDCs implanted onto NuOss™ calcium phosphate scaffolds in a murine *in vivo *model.

## Results

Circulating mononuclear precursors were increased in patients compared to healthy controls (99.1% vs. 83.9%; *P *< 0.05). Patient PBMCs differentiated into mature actively resorbing osteoclasts in the presence or absence of osteoclastogenic factors (3.2-fold increase vs. healthy cells; *P *< 0.01) and when cultured in PS this spontaneous osteoclast formation was increased further (2.3-fold; *P *< 0.05). There were no differences in the osteogenic differentiation of hPDCs treated with PS, but there was a twofold (*P *< 0.01) decrease in vascular endothelial growth factor receptor 1 expression. Scaffolds with patient serum-treated hPDCs displayed decreased vascularization and increased osteoclast activity leading to a 28.9% (*P *< 0.001) decrease in bone formation.

## Conclusion

Circulating mononuclear precursors from critically ill patients seem prone to form osteoclasts both in the presence of osteoclastogenic factors and spontaneously. The murine *in vivo *model confirmed an increase in osteoclastic resorption and a decreased vascularization, leading to decreased bone formation in patient scaffolds. These findings will help to unravel the mechanisms behind bone loss during critical illness.

